# Monitoring skin blood flow to rapidly identify alterations in tissue perfusion during fluid removal using continuous veno-venous hemofiltration in patients with circulatory shock

**DOI:** 10.1186/s13613-021-00847-z

**Published:** 2021-04-14

**Authors:** Wasineenart Mongkolpun, Péter Bakos, Jean-Louis Vincent, Jacques Creteur

**Affiliations:** Department of Intensive Care, Erasme University Hospital, Université Libre de Bruxelles, Route de Lennik 808, 1070 Brussels, Belgium

**Keywords:** Peripheral perfusion, Microcirculation, Hemodialysis, Laser flowmetry, Lactate concentration

## Abstract

**Background:**

Continuous veno-venous hemofiltration (CVVH) can be used to reduce fluid overload and tissue edema, but excessive fluid removal may impair tissue perfusion. Skin blood flow (SBF) alters rapidly in shock, so its measurement may be useful to help monitor tissue perfusion.

**Methods:**

In a prospective, observational study in a 35-bed department of intensive care, all patients with shock who required fluid removal with CVVH were considered for inclusion. SBF was measured on the index finger using skin laser Doppler (Periflux 5000, Perimed, Järfälla, Sweden) for 3 min at baseline (before starting fluid removal, T0), and 1, 3 and 6 h after starting fluid removal. The same fluid removal rate was maintained throughout the study period. Patients were grouped according to absence (Group A) or presence (Group B) of altered tissue perfusion, defined as a 10% increase in blood lactate from T0 to T6 with the T6 lactate ≥ 1.5 mmol/l. Receiver operating characteristic curves were constructed and areas under the curve (AUROC) calculated to identify variables predictive of altered tissue perfusion. Data are reported as medians [25th–75th percentiles].

**Results:**

We studied 42 patients (31 septic shock, 11 cardiogenic shock); median SOFA score at inclusion was 9 [8–12]. At T0, there were no significant differences in hemodynamic variables, norepinephrine dose, lactate concentration, ScvO_2_ or ultrafiltration rate between groups A and B. Cardiac index and MAP did not change over time, but SBF decreased in both groups (*p* < 0.05) throughout the study period. The baseline SBF was lower (58[35–118] vs 119[57–178] perfusion units [PU], *p* = 0.03) and the decrease in SBF from T0 to T1 (ΔSBF%) higher (53[39–63] vs 21[12–24]%, *p* = 0.01) in group B than in group A. Baseline SBF and ΔSBF% predicted altered tissue perfusion with AUROCs of 0.83 and 0.96, respectively, with cut-offs for SBF of ≤ 57 PU (sensitivity 78%, specificity 87%) and ∆SBF% of ≥ 45% (sensitivity 92%, specificity 99%).

**Conclusion:**

Baseline SBF and its early reduction after initiation of fluid removal using CVVH can predict worsened tissue perfusion, reflected by an increase in blood lactate levels.

**Supplementary Information:**

The online version contains supplementary material available at 10.1186/s13613-021-00847-z.

## Introduction

Optimal fluid balance is an essential part of patient management. In patients with circulatory shock, fluid administration is widely used to increase cardiac output and restore tissue perfusion, but a high fluid balance can result in tissue edema and is associated with increased mortality [1–4]. When spontaneous diuresis is inadequate, fluid removal using continuous veno-venous hemodialysis (CVVH) is a valuable therapeutic option to eliminate excess fluid [4, 5]. However, aggressive fluid removal can cause hemodynamic compromise resulting in tissue hypoperfusion, and necessitating cessation of fluid removal despite the persistence of tissue edema [2, 3, 6–8]. In severe cases, hypotension may develop, leading to impaired organ function and increased mortality [9–11].

At the bedside, decreased tissue perfusion can be assessed by an abnormally elevated blood lactate concentration, although several medications, such as metformin, propofol or β2 agonists, can also influence lactate levels [12, 13]. The persistence of hyperlactatemia or an increase in lactate concentration during resuscitation reflects the severity of tissue hypoperfusion and is related to more severe organ dysfunction and higher mortality [14–17]. Hyperlactatemia usually presents when hemodynamic conditions are compromised [18–20].

Skin hypoperfusion occurs before systemic hemodynamic variables worsen [9, 21–23] and its persistence has been shown to be associated with higher mortality [24–26]. Additionally, a shortening of the prolonged capillary refill time (CRT) during resuscitation suggested resolution of tissue hypoperfusion, as reflected by a reduction in lactate concentration [25, 27–29]. Hence, monitoring changes in skin perfusion may be useful to track the development or worsening of tissue hypoperfusion.

Skin laser Doppler (SLD) is a simple noninvasive tool that has been widely used in clinical studies to evaluate cutaneous microcirculatory perfusion [24, 26, 30]. SLD measures local microcirculatory blood flow including perfusion in capillaries (nutritive flow), arterioles, venules and shunting vessels [31–33]. We recently showed that skin blood flow (SBF) measured on the finger using SLD was altered in circulatory shock [26].

In the present study, we evaluated changes in SBF during fluid removal with CVVH in patients with circulatory shock and determined whether changes were associated with altered tissue perfusion as reflected by an increase in blood lactate concentration.

## Methods

This prospective, observational study was conducted from 1 November 2017 to 30 October 2018 in the 35-bed Department of Intensive Care of Erasme University Hospital (Brussels, Belgium). The study protocol was approved by the local ethical Committee (Protocol number P2017/013/B406201730812), and informed consent was signed by all patients or their next of kin.

### Patients

All consecutive ICU patients with circulatory shock (vasopressor support needed to maintain mean arterial pressure [MAP] ≥ 65 mmHg, and at least one sign of poor tissue perfusion [altered conscious level, mottled skin, or arterial lactate ≥ 2 mmol/l] [34–36] were screened. If, during the ICU stay, these patients required fluid removal using continuous veno-venous hemodiafiltration (CVVHDF), they were considered for inclusion. Patients with any of the following were excluded: presence of any skin lesion at the site of measurement that would have made measurements difficult; a history of Raynaud's phenomenon or systemic sclerosis; receipt of metformin, propofol, β2 agonist or a blood transfusion during the study period; refusal to sign informed consent; previous inclusion in the study.

### Measurements

The APACHE II score [37] was calculated using the worst data during the first 24 h following ICU admission. The sequential organ failure assessment (SOFA) score [38] on admission and at the time of study inclusion (start of fluid removal using CVVHDF) were recorded. The presence of signs of poor tissue perfusion [oliguria (urine output < 0.5 ml/kg/h), altered conscious level assessed using the “assumed” Glasgow Coma Scale, mottled skin, arterial lactate level ≥ 2 mmol/l] were noted at shock diagnosis and the CRT was measured (routine practice in patients with shock during the study period); the same variables were recorded at study inclusion (start of fluid removal using CVVHDF). Skin mottling was assessed using the mottling score [39] and a score ≥ 2 was considered as clinically significant. CRT was determined by applying pressure to the tip of the finger for at least 15 s until the skin showed whitening; the time until return of baseline coloration after release of the pressure was measured with a chronometer.

SBF was measured on the ventral side of the index finger at study inclusion (T0), and 1 (T1), 3 (T3), and 6 (T6) hours later. The researchers who performed the SLD measurements were different from the team managing the CVVHDF. The peripheral perfusion index (PPI) was obtained from the pulse oximeter positioned on the contralateral side to that used for SBF measurements and recorded from the bedside monitor (IntelliVue MP70 monitor, Philips Medical Systems, Boblingen, Germany) at the same timepoints.

Hemodynamic variables, norepinephrine dose and blood gas data, including central venous oxygen saturation (ScvO_2_) and arterial lactate concentration, were obtained at shock diagnosis and at T0, T1, T3 and T6. The use of mechanical ventilation at the time of shock diagnosis, and the duration of CVVHDF and the cumulative fluid balance from the onset of circulatory shock until study inclusion were recorded. The use of sedative agents during the 24 h after ICU admission and at T0 was noted. If the norepinephrine dose was increased during the study period, the time from T0 to the first increase in norepinephrine dose was noted; the MAP just before the first increase in norepinephrine dose was also recorded.

### SBF measurements

SBF was evaluated using a SLD device (PeriFlux System 5000, Perimed, Jarfalla, Sweden) with a small thermostatic SLD probe (Reference number 457, Perimed). This probe enables one to simultaneously measure and alter the temperature at the place where it is positioned. The probe is attached to the skin with a double-sided tape provided with the monitor. The laser beam emitted by the SLD machine has a wavelength of 780 nm, which allows an evaluation depth between 0.5 and 1.0 mm below the skin surface. The back-scattered light is collected by the probe and the change in light wavelength is proportional to the red blood cell (RBC) velocity in the studied area, thus providing a noninvasive measurement of SBF expressed as perfusion units (PU). Data were continuously recorded for future off-line analyses using PeriSoft software 2.5.5 (Perimed). To reduce the short-term intra-individual variation in SLD that has been reported in previous studies [40–42], the SLD probe was kept on the same area in each patient throughout the study period. The relative change in SBF (∆SBF%) during the first hour of fluid removal by CVVHDF was calculated using the formula: SBF at T0−SBF at T1/ SBF at T0 × 100. All SBF measurements were obtained in the supine position and at basal skin temperature, which was recorded prior to the measurement. During the measurements, hand movements and changes to the patient’s position were not allowed; SBF measurements were delayed at least 10 min in agitated patients.

### Renal replacement therapy

All patients who underwent fluid removal by CVVHDF (Primaflex^®^, Baxter, Chicago, USA) with a ST100-AN69 membrane had a 14F double-lumen catheter inserted in the femoral or jugular vein. According to our local protocol, blood flow and dialysate flow were set at 2 ml/min/kg and 20 ml/h/kg, respectively. Pre-dilution and post-dilution replacement fluids were prescribed at 20 ml/h/kg and 10 ml/h/kg, respectively. Regional anticoagulation was achieved using trisodium citrate. Arterial blood gases and ionized calcium concentration were evaluated and corrected according to local protocols. The time to initiate fluid removal by CVVHDF and the rate of fluid removal were determined by the care team; the rate was kept stable during the study period.

## Statistical analysis

The normality of the distribution in all variables was tested using a Kolmogorov–Smirnov test and data are presented as median (25th–75th percentiles) or mean (with standard deviation) as appropriate. Patients were separated into two groups according to the absence (group A) or presence (group B) of altered tissue perfusion, defined as an increase in blood lactate of  ≥ 10% over 6 h (with a 6-h lactate ≥ 1.5 mmol/l). Additionally, the patients were grouped by the type of shock (septic shock vs cardiogenic shock) and the start of fluid removal by CVVHDF (fluid removal started at the same time as CVVHDF vs fluid removal started after initiation of CVVHDF). Differences between groups were assessed using a Chi-square, Fisher’s exact test, Mann–Whitney *U* test, ANOVA with Bonferroni post hoc analysis or Kruskal–Wallis test as appropriate. Repeated ANOVA was used to evaluate the change in SBF over time between groups. We plotted the sensitivity and specificity using a receiving operating characteristics (ROC) curve, and the area under the curve (AUROC) was calculated for the different variables as a measure of their ability to predict altered tissue perfusion. AUROCs are presented as mean ± SD with 95% confidence interval and were compared using the Hanley and McNeil method. To assess possible variables correlated with the different SBF-derived variables, we plotted individual data on graphs and calculated the Pearson or Spearman correlation coefficient (*r*^2^) as appropriate. A two-sided *p*-value < 0.05 was considered as statistically significant. All analyses were performed using STATA15.0 (Stata Corp LLC, College Station, Texas).

## Results

Eighty-two patients with circulatory shock were screened; 53 underwent fluid removal by CVVHDF and were considered for inclusion. Eleven were excluded, because of severe agitation (*n* = 1), receipt of blood transfusion (*n* = 3), and change in ultrafiltration rate during the study period (*n* = 7) so that 42 were included in the study (31 with septic shock, 11 with cardiogenic shock). Fluid removal was initiated at the same time as CVVHDF in 19 patients, and a median of 3.5 (2.4–5.7) hours after CVVHDF had been started in 23 patients. Tissue perfusion was altered (as defined by an increase in blood lactate of  ≥ 10% over 6 h, with a 6-h lactate ≥ 1.5 mmol/l) in 22 patients (Group B) and unaltered in 20 (Group A).

There were no differences in severity scores, MAP, cardiac index (CI), norepinephrine dose, lactate concentration, presence of oliguria, or CRT between groups A and B at the time of diagnosis of shock (Additional file 1: Table S1). Skin mottling was present in seven patients in Group B and none in Group A at shock diagnosis (Additional file 1: Table S1). The median lactate concentration in all patients was 3.0 (2.8–3.8) mmol/l (Additional file 1: Table S1).

At study inclusion, there were no significant differences between groups A and B in severity scores, MAP, CI, norepinephrine dose, lactate concentration, ScvO_2_, PPI, CRT, fluid removal rate, or cumulative fluid balance (Table 1) or between septic shock versus cardiogenic shock patients (Additional file 1: Table S2). Norepinephrine dose was lower in patients whose fluid removal was started at the same time as CVVHDF than in patients whose fluid removal started later (Additional file 1: Table S3). Oliguria was observed in all patients at study inclusion and hyperlactatemia in 11 patients (Table 1). No patients had skin mottling at study inclusion. Sixteen patients (38%) died in the ICU.

**Table 1:** Main clinical characteristics at admission or study inclusion

Characteristic	All patients (*n* = 42)	Group A (*n* = 20)	Group B (*n* = 22)	*p* value
On admission				
Age (years)	63 (53–73)	68 (48–73)	61 (57–71)	0.6
APACHE II score	24 (17–26)	22 (14–27)	24 (17–26)	0.8
SOFA score	12 (9–14)	11 (8–14)	10 (10–14)	0.8
At study inclusion				
SOFA score	9 (8–12)	10 (9–12)	9 (7–10)	0.1
Mean arterial pressure (mmHg)	77 (74–87)	78 (76–86)	76 (74–90)	0.3
Heart rate (bpm)	97 (86–109)	99 (81–108)	97 (86–112)	0.5
Cardiac index (L/min/m^2^) (*n* = 27)	3.1 (2.9–4.1)	3.1 (2.9–3.2)	3.0 (2.7–4.0)	0.6
Norepinephrine dose (mcg/kg/min) (*n* = 34)	0.2 (0.1–0.5)	0.2 (0.2–0.5)	0.2 (0.1–0.6)	0.3
Sedation, *n* (%)	19 (45%)	6 (30%)	13 (59%)	0.2
Sufentanil (mcg/kg/h)	1.4 (1.3–1.6)	1.3 (1.3–1.4)	1.5 (1.2–1.8)	0.4
Midazolam (mg/kg/h)	0.02 (0.01–0.03)	0.02 (0.01–0.02)	0.02 (0.02–0.03)	0.8
PaO_2_/FiO_2_	197 (161–265)	172 (132–221)	198 (178–282)	0.1
Central venous pressure (mmHg)	11 (8–14)	11 (8–13)	12 (7–15)	0.9
Lactate concentration (mmol/l)	1.5 (1.2–2.1)	1.5 (1.2–2.1)	1.4 (1.2–2.1)	0.6
Lactate concentration > 2 mmol/l, *n* (%)	11 (26)	5 (25)	6 (26)	0.7
Oliguria (urine output < 0.5 ml/kg/h), *n* (%)	42 (100)	20 (100)	22 (100)	0.8
ScvO_2_ (%)	71 (69–73)	71 (69–73)	70 (68–72)	0.8
Capillary refill time (seconds)	2.2 (2.0–2.7)	2.2 (2.0–2.5)	2.4 (2.0–2.9)	0.4
Hemoglobin level (g/dl)	9 (7–11)	10 (9–11)	9 (8–11)	0.3
Peripheral perfusion index (PPI)	2 (0.8–2.3)	1.7 (1.4–2.1)	2.1 (0.8–2.3)	0.6
Fluid removal rate (ml/h)	100 (100–200)	100 (100–200)	150 (100–200)	0.1
Time between diagnosis of shock and start of fluid removal by CVVHDF (hours)	34 (24–49)	45 (36–50)	26 (14–48)	0.06
Fluid removal duration (days)	10 (8–12)	8 (8–10)	10 (10–12)	0.1
Cumulative fluid balance before start of fluid removal by CVVHDF (ml)	2404 (1368–3777)	3211 (2026–4000)	2235 (1368–3289)	0.2
Mechanical ventilation, *n* (%)	22 (52)	7 (35)	15 (68)	0.03

Data are expressed as median with 25th and 75th percentile unless otherwise specified

*APACHE II* Acute Physiology and Chronic Health Evaluation II, *CVVDHF* continuous veno-venous hemodiafiltration, *ScvO*_*2*_ central venous oxygen saturation, *SOFA* Sequential Organ Failure Assessment

### Hemodynamic and tissue oxygenation parameters during fluid removal with CVVHDF

MAP, CI, ScvO_2_, PPI and finger temperature did not change significantly during the study period in all patients (Table 2). Norepinephrine dose was higher in group B than in group A (*p* = 0.02) at T6, and lactate concentration (*p* = 0.03) increased during the study period only in group B (Table 2). The median time from T0 to the first increase in norepinephrine dose was 120 (70–145) minutes in group B. The MAP at the time of first increase in norepinephrine dose was 16 (10–27)% lower than at baseline.

**Table 2:** Hemodynamic parameters during the study period in patients with (group B) and without (group A) altered tissue perfusion

Parameter	T0	T1	T3	T6
MAP (mmHg)				
Group A	78 (76–86)	76 (72–86)	72 (70–83)	78 (75–82)
Group B	76 (74–90)	73 (68–79)	74 (72–86)	75 (72–87)
CI (L/min/m^2^) (*n* = 27)				
Group A	3.1 (2.9–3.2)	3.2 (3.0–3.3)	3.1 (2.9–3.5)	3.2 (2.7–3.6)
Group B	3.0 (2.7–4.0)	2.9 (2.7–4.1)	3.0 (2.7–4.0)	3.1 (2.9–3.8)
Norepinephrine dose (mcg/kg/min) (*n* = 34)				
Group A	0.2 (0.2–0.5)	0.2 (0.2–0.4)	0.2 (0.1–0.3)	0.2 (0.2–0.3)
Group B	0.2 (0.1–0.6)	0.2 (0.1–0.7)	0.3 (0.1–0.6)	0.4 (0.3–0.8)^#^*
Lactate concentration (mmol/l)				
Group A	1.5 (1.2–2.1)	1.5 (1.2–2.3)	1.5 (1.3–2.0)	1.4 (1.3–1.6)
Group B	1.4 (1.2–2.1)	1.5 (1.3–2.0)	1.7 (1.5–2.1)	2.2 (1.8–2.7)^#^*
ScvO_2_ (%)				
Group A	71 (69–72)	70 (69–74)	71 (68–72)	70 (68–71)
Group B	70 (68–71)	71 (69–73)	70 (68–74)	69 (67–71)
PPI				
Group A	1.7 (1.4–2.1)	1.6 (1.2–1.9)	1.9 (1.5–2.2)	2.1 (1.3–2.2)
Group B	2.1 (0.8–2.3)	1.6 (0.9–2.1)	1.8 (1.4–2.2)	1.5 (1.2–2.1)

Data are expressed as medians with 25th and 75th percentiles

*CI* cardiac index, *MAP* mean arterial pressure, *PPI* peripheral perfusion index, *ScvO*_*2*_, central venous oxygen saturation

*p < 0.05 between groups A and B at that time point

^#^*p* < 0.05 versus baseline (T0) value in the same group

### Change in SBF during fluid removal with CVVHDF

**Fig. 1:**
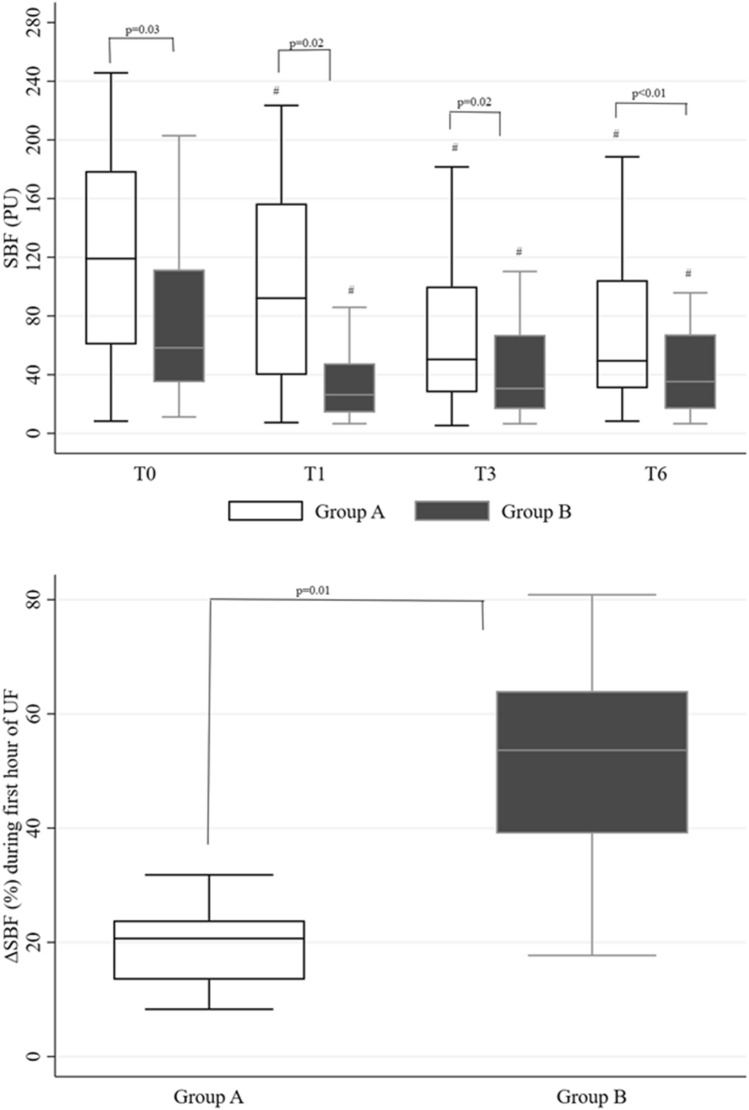
Box-and-whisker plots (median and interquartile range) of skin blood flow (SBF) during fluid removal with CVVHDF (**a**) and change in SBF (∆SBF) during the first hour of fluid removal with CVVHDF (UF; **b**) in the two groups. ^#^*p* < 0.05 vs baseline (T0) in the same group; *PU* perfusion unit

SBF was lower at all time points in group B than in group A (Fig. 1a). SBF decreased in both groups from T0 to T1 (Fig. 1a), but the ∆SBF% during this time period was higher in patients in group B than group A (*p* = 0.01) (Fig. 1b). SBF was similar in patients with septic shock and cardiogenic shock (Additional file 1: Table S2), and decreased similarly in both groups from T0 to T1 (∆SBF% 32 [15–54]% vs 30 [20–63]%, *p* = NS). Changes in SBF from T0 to T1 were similar in patients with fluid removal starting at initiation of CVVHDF and those with fluid removal starting later (∆SBF% 28 [18–58]% vs 32 [15–48]%, *p* = NS) (Additional file 1: Table S3).

**Fig. 2:**
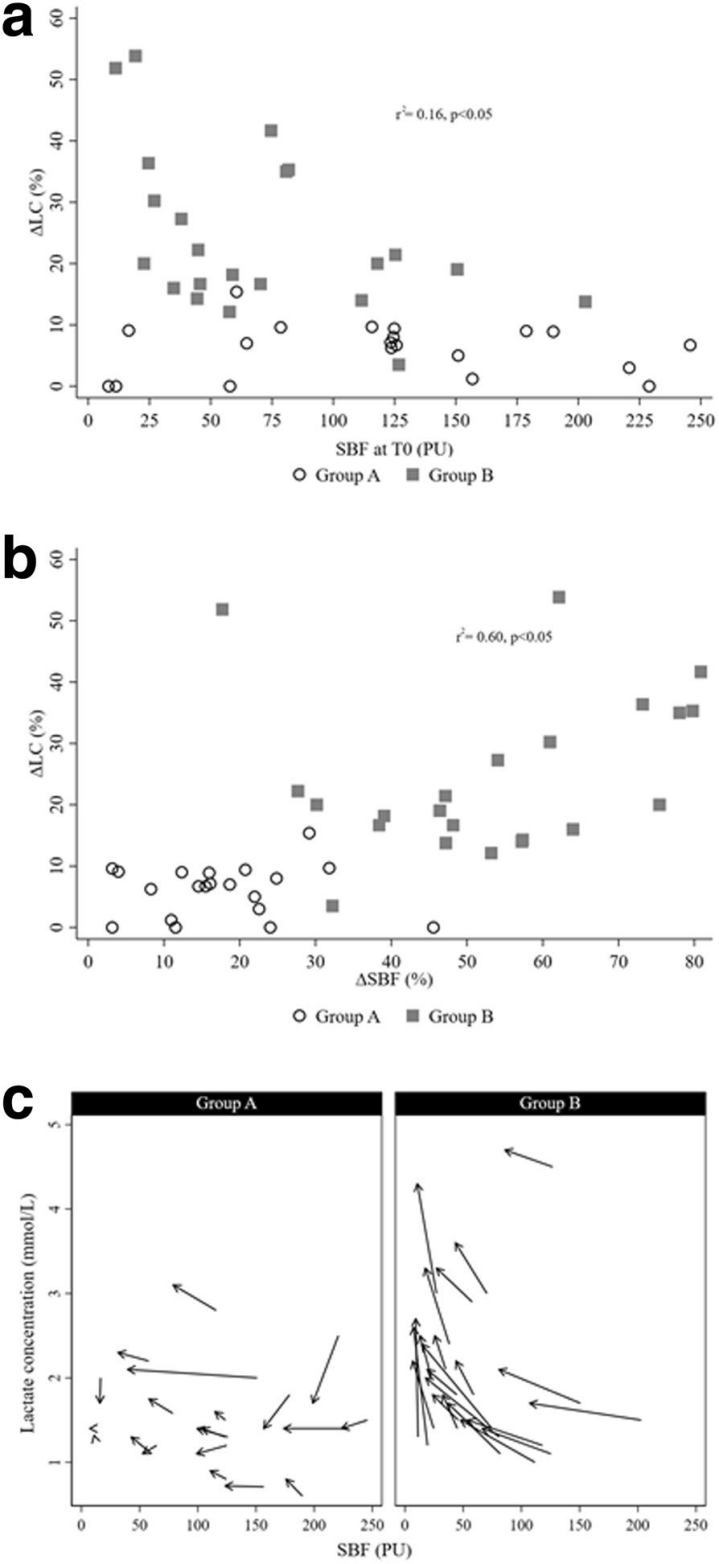
Correlations between change in lactate concentration (∆LC%) and **a** skin blood flow (SBF) at baseline (T0) and **b** change in SBF over first hour of fluid removal with CVVHDF (∆SBF%); **c** individual change in SBF and lactate concentration from baseline to 6 h after start of fluid removal with CVVHDF in groups A and B

The SBF at T0 (*r*^2^ = 0.16, *p* = 0.04) (Fig. 2a) and the ∆SBF% throughout the study period (*r*^2^ = 0.60, *p* < 0.01) were correlated with the increase in blood lactate concentration during the first 6 h of fluid removal (Fig. 2b). Individual changes in SBF and lactate concentration from the first hour of fluid removal by CVVDHF to the 6th hour are shown in Fig. 2c.

### Variables to predict the presence of altered tissue perfusion

**Fig. 3:**
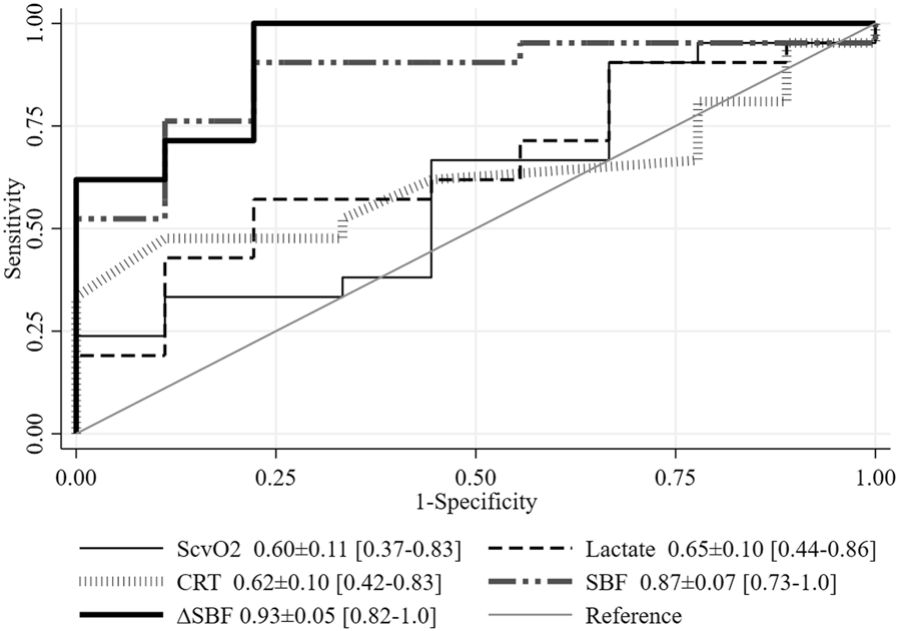
Areas under the curve for prediction of change in tissue perfusion (change in lactate concentration ≥ 10% with lactate concentration at T6 ≥ 1.5 mmol/l). *ScvO*_*2*_ central venous oxygen saturation, *CRT* capillary refill time, *SBF* skin blood flow, ∆*SBF* change in SBF from T0–T1

The AUROCs for SBF at T0 (0.87 ± 0.07 [0.73–1.0]) and the ∆SBF% from T0 to T1 (0.93 ± 0.05 [0.82–1.0]) were higher than the AUROCs for other variables (*p* = 0.01), with cut-off points of ≤ 57 PU (sensitivity 78%, specificity 87%) and ≥ 45% (sensitivity 92%, specificity 99%), respectively (Fig. 3); the AUROC for baseline SBF was similar to that of ∆SBF%. The AUROCs for CI, MAP and norepinephrine dose at T0 were 0.55 ± 0.13 [0.28–0.82], 0.64 ± 0.12 [0.41–0.88], and 0.65 ± 0.13 [0.38–0.92], respectively.

## Discussion

In this prospective observational study, SBF at the start of fluid removal using CVVHDF and a decrease in SBF during the first hour of fluid removal were both related to a worsening of tissue perfusion as measured by an increase in lactate concentration at 6 h compared to baseline. Additionally, despite a reduction in SBF during fluid removal by CVVHDF, MAP, CI, PPI and ScvO_2_ did not change during the study period.

In patients with circulatory shock, increased blood lactate concentration is an indicator of altered tissue perfusion [17, 43, 44] and is associated with increased morbidity and mortality [15, 16, 36, 43, 45], including in patients with septic shock [16, 36, 43–46]. Even a mildly elevated lactate concentration, ≥ 1.5 mmol/l, is associated with worse outcomes [16, 47, 48] and related to altered sublingual microcirculatory perfusion [16]. Moreover, dynamic changes in lactate levels during resuscitation are as important as absolute lactate values [43, 49]. A reduction in lactate concentration during the resuscitation period is associated with decreased mortality [44, 50–52], whereas increasing lactate concentrations suggest that treatment should be reassessed [49]. Although the optimal cut-off points for decrease in lactate are debated, Nguyen et al. [14] reported that a lactate reduction ≥ 10% over a 6-h period was related to improved survival, and Jones et al. [53] used a lactate reduction > 10% as a target for resuscitation in their randomized controlled trial in patients with sepsis. In the present study, we therefore chose to define worsening tissue perfusion as an increase in blood lactate concentration of ≥ 10% during a 6-h period, with a T6 lactate of at least 1.5 mmol/l. The change in lactate concentration in patients with altered tissue perfusion may be explained by the occurrence of hemodynamic compromise, as shown by an increase in norepinephrine dose in these patients during the study period.

The stable CI, MAP and ScvO_2_, despite the decrease in SBF, suggest that finger SBF was more sensitive than systemic hemodynamic variables and ScvO_2_ for detecting impaired tissue perfusion. ScvO_2_ did not decrease during fluid removal by CVVHDF as was reported by Zhang et al. [54]. This observation may be explained by a higher ultrafiltration rate in that study [54] compared to ours [1900 ± 800 ml in 3.65 h vs 600 (450–1200) ml in 6 h, respectively]. The PPI also did not decrease in our study as was reported in a study by Klijn et al. [55]. This might again be explained by the different fluid removal rates: in the present study, the fluid removal rate was fixed during the study period, whereas in the study by Klijn et al. [55] it was doubled every 15 min. Bigé et al. [56] observed that an index CRT > 3 s had a reasonably good predictive value for intradialytic hypotension, but this measure was less reliable in our patients (AUC 0.62 vs 0.71), perhaps because we included fewer patients with a CRT > 3 s at baseline (27% vs 55%, respectively).

SBF was lower throughout the study period in patients with altered tissue perfusion. One may argue that the lower SBF in these patients was caused by the higher norepinephrine dose. However, several studies using muscle tissue oxygenation [57] and sublingual microcirculatory assessment [57–59] have shown that norepinephrine does not reduce peripheral tissue perfusion. Moreover, in a recent study [26], we showed that finger SBF, obtained using SLD, was not related to norepinephrine dose in patients with circulatory shock. Therefore, a lower SBF in patients with altered tissue perfusion during the study period was likely to have been associated with the fluid removal process rather than the higher vasopressor doses. Of note, changes in SBF were not more reliable than single SBF values, but these two parameters could guide fluid removal therapy in distinct ways. Patients with higher SBF at baseline appear to have a low risk of developing hemodynamic compromise, and measurement of SBF could thus help physicians select those patients who are suitable for fluid removal therapy. By contrast, the ∆SBF may help adjust the fluid removal rate. From our results, a reduction in SBF of  ≥ 45% during the first hour after fluid removal is started should prompt a reduction in the fluid removal rate. Therefore, measuring the SBF at baseline and monitoring the change in SBF over time could help prevent worsening of tissue hypoperfusion during fluid removal by CVVHDF.

The monocenter nature of this study is both an advantage and a limitation. An advantage is the relative homogeneity in procedures. A limitation is that our data may not apply to other units with other protocols in place. Additionally, because of the criteria used to define altered tissue perfusion, we excluded patients who had received propofol because of the potential effect of propofol infusion on lactate concentration [12, 60]. Our observations need to be validated in multicenter studies including larger sample sizes.

## Conclusion

A lower SBF at the start of fluid removal using CVVHDF and reduction in SBF over the first hour of treatment are related to the development of new tissue hypoperfusion.

## Supplementary Information

**Additional file 1: Table S1. **Main characteristics in patients without (group A) and with (group B) altered tissue perfusion at the time of diagnosis of shock. **Table S2.** Hemodynamic variables and skin blood flow (SBF) during the study period in patients with septic shock and cardiogenic shock. **Table S3.** Hemodynamic variables and skin blood flow (SBF) during the study period in patients in whom fluid removal was started at the same time as continuous veno-venous hemodiafiltration (CVVHDF) or later

## Data Availability

The datasets used during the current study are available from the corresponding author on reasonable request.

## Abbreviations

CI: Cardiac index
CVVH: Continuous veno-venous hemofiltration
CVVHDF: Continuous veno-venous hemodiafiltration
CRT: Capillary refill time
CVP: Central venous pressure
MAP: Mean arterial pressure
PPI: Peripheral perfusion index
SBF: Skin blood flow
SLD: Skin laser Doppler
ScvO_2_: Central venous oxygen saturation
RBC: Red blood cell
