# Supplementary material for: Monitoring skin blood flow to rapidly identify alterations in tissue perfusion during fluid removal using continuous veno-venous hemofiltration in patients with circulatory shock
Source: Ann Intensive Care. 2021 Apr 14;11:59. doi: 10.1186/s13613-021-00847-z (PMC8046875; doi:10.1186/s13613-021-00847-z)
Supplement: Supplementary file 1 — Additional file 1: Table S1. Main characteristics in patients without (group A) and with (group B) altered tissue perfusion at the time of diagnosis of shock. Table S2. Hemodynamic variables and skin blood flow (SBF) during the study period in patients with septic shock and cardiogenic shock. Table S3. Hemodynamic variables and skin blood flow (SBF) during the study period in patients in whom fluid removal was started at the same time as continuous veno-venous hemodiafiltration (CVVHDF) or later [file 13613_2021_847_MOESM1_ESM.docx]

| **Variable** | **All patients**  **(N=42)** | **Group A**  **(N=20)** | **Group B**  **(N=22)** | **p** |
| --- | --- | --- | --- | --- |
| Type of shock   - Septic shock, n (%) - Cardiogenic shock, n (%) | 31 (73)  11 (26) | 16 (72)  6 (27) | 15 (75)  5 (25) | 0.7 |
| Sign of poor tissue perfusion, n (%)  Oliguria (urine output <0.5 ml/kg/hour)  Mottled skin (mottling score ≥ 2)  Altered level of consciousness | 26 (62)  7 (16)  6 (14) | 12 (60)  0 (0)  2 (10) | 14 (63)  7 (32)  4 (18) | 0.6  0.01  0.5 |
| Mean arterial pressure (mmHg) | 75 (67-82) | 73 (67-83) | 78 (67-87) | 0.8 |
| Heart rate (bpm) | 95 (75-104) | 92 (76-100) | 95 (75-101) | 0.7 |
| Cardiac index (L/min/m^2^) (n=24) | 2.4 (2.2-3.0) | 2.3 (2.2-2.9) | 2.5 (2.2-3.0) | 0.4 |
| Norepinephrine dose (mcg/kg/min) (n=42) | 0.3 (0.2-0.4) | 0.2 (0.2-0.4) | 0.3 (0.2-0.7) | 0.7 |
| Patients receiving sedation during first 24 hours of ICU admission (%)  - Sufentanil (mcg/kg/hour) (n=13)  - Midazolam (mg/kg/hour) (n=8) | 21 (50%)  1.5 (0.9-2.0)  0.03 (0.01-0.03) | 8 (40%)  1.5 (1.2-2.0)  0.02 (0.01-0.03) | 13 (59%)  1.4 (1.0-2.0)  0.02 (0.02-0.03) | 0.4  0.9  0.8 |
| ScvO_2_ (%) | 70 (67-72) | 69 (67-72) | 70 (67-72) | 0.5 |
| Lactate concentration (mmol/L) | 3.0 (2.8-3.8) | 2.9 (2.7-3.3) | 3.1 (2.9-4.3) | 0.2 |
| Capillary refill time (seconds) | 3.8 (3.2-4.7) | 3.7 (2.7-4.8) | 3.8 (3.2-4.7) | 0.6 |
| Mechanical ventilation, n (%) | 22 (52) | 7 (35) | 15 (68) | 0.03 |

**Monitoring skin blood flow to rapidly identify alterations in tissue perfusion during ultrafiltration in patients with circulatory shock**

Mongkolpun W et al

**Additional file 1**

**Table S1** Main characteristics in patients without (group A) and with (group B) altered tissue perfusion at the time of diagnosis of shock

Data are expressed as medians with 25th and 75th percentile or number (%)

Abbreviations: APACHE II-Acute Physiology And Chronic Health Evaluation II; SOFA-Sequential Organ Failure Assessment; ScvO_2_-central venous oxygen saturation

**Table S2** Hemodynamic variables and skin blood flow (SBF) during the study period in patients with septic shock and cardiogenic shock

| Variable | Group | T0 | T1 | T3 | T6 |
| --- | --- | --- | --- | --- | --- |
| MAP (mmHg) | Septic shock (n=31) | 77 (74-86) | 72 (68-86)^#^ | 74 (72-86) | 77 (72-86) |
|  | Cardiogenic shock (n=11) | 78 (74-90) | 78 (72-86) | 72 (70-90)^#^ | 79 (72-94) |
| CI (L/min/m^2^) | Septic shock (n=21) | 3.1 (2.8-3.4) | 3.0 (2.7-3.5) | 3.1 (2.8-3.6) | 3.1 (2.8-3.7) |
|  | Cardiogenic shock (n=6) | 3.0 (2.9-3.2) | 3.1 (2.8-3.2) | 3.0 (2.9-3.3) | 3.0 (2.8-3.2) |
| Norepinephrine dose (mcg/kg/min) | Septic shock (n=27) | 0.2 (0.2-0.7)^*^ | 0.2 (0.2-0.7)^*^ | 0.3 (0.2-0.7)^*^ | 0.4 (0.2-0.8)^#,*^ |
|  | Cardiogenic shock (n=7) | 0.1 (0.02-0.10) | 0.1 (0.05-0.20) | 0.1 (0.05-0.01) | 0.2 (0.1-0.30) |
| Lactate concentration (mmol/L) | Septic shock (n=31) | 1.5 (1.2-2.0) | 1.5 (1.3-2.4) | 1.5 (1.3- 2.4) | 1.7 (1.4-2.6)^#^ |
|  | Cardiogenic shock (n=11) | 1.8 (1.3-2.1) | 1.9 (1.4-1.9) | 1.8 (1.4-2.1) | 1.9 (1.5-2.3) |
| ScvO_2_ (%) | Septic shock (n=31) | 71 (70-75) | 71 (69-72) | 70 (68-75) | 70 (69-72) |
|  | Cardiogenic shock (n=11) | 71 (71-74) | 71 (69-72) | 69 (68-72) | 70 (67-72) |
| SBF (PU) | Septic shock (N=31) | 79 (37-125) | 43 (16-85)^#^ | 40 (17-86)^#^ | 45 (17-74)^#^ |
|  | Cardiogenic shock (n=11) | 81 (45-156) | 38 (26-99)^#^ | 39 (31-49)^#^ | 46 (35-52)^#^ |

Data are expressed as median with 25th and 75th percentile, *p<0.05 septic shock vs cardiogenic shock, #p<0.05 compared to baseline value (T0) in the same group

Abbreviations: MAP-mean arterial pressure; CI-cardiac index; ScvO_2_-central venous oxygen saturation; PU: perfusion unit

**Table S3** Hemodynamic variables and skin blood flow (SBF) during the study period in patients in whom fluid removal was started at the same time as continuous veno-venous hemodiafiltration (CVVHDF) or later

| **Variable** | **Group** | **T0** | **T1** | **T3** | **T6** |
| --- | --- | --- | --- | --- | --- |
| **MAP (mmHg)** | Fluid removal started at initiation of CVVHDF (N=19) | 76 (74-79) | 75 (69-78) | 72 (70-80) | 75 (72-83) |
|  | Fluid removal started after initiation of CVVHDF (N=23) | 78 (74-90) | 77 (69-87) | 75 (72-88) | 78 (74-88) |
| **CI (L/min/m^2^)** | Fluid removal started at initiation of CVVHDF (N=12) | 3.2 (2.8-3.3) | 3.1 (2.8-3.4) | 3.2 (2.8-3.4) | 3.2 (2.9-3.5) |
|  | Fluid removal started after initiation of CVVHDF (N=15) | 3.0 (2.8-3.7) | 3.1 (2.8-3.8) | 3.0 (2.9-3.9) | 3.1 (2.8-3.8) |
| **Norepinephrine dose (mcg/kg/min)** | Fluid removal started at initiation of CVVHDF (N=14) | 0.1 (0.05-0.2)* | 0.1 (0.1-0.2)* | 0.2 (0.1-0.3)* | 0.3 (0.2-0.5)# |
|  | Fluid removal started after initiation of CVVHDF (N=20) | 0.3 (0.2-0.7) | 0.3 (0.2-0.8) | 0.3 (0.2-0.8) | 0.4 (0.2-0.7) |
| **Lactate concentration**  **(mmol/L)** | Fluid removal started at initiation of CVVHDF (N=19) | 1.4 (1.3-2.1) | 1.6 (1.3-2.1) | 1.7 (1.3-2.2) | 2.0 (1.5-2.5)# |
|  | Fluid removal started after initiation of CVVHDF (N=23) | 1.5 (1.2-2.0) | 1.5 (1.3-2.1) | 1.5 (1.4-2.1) | 1.7 (1.4-2.2)# |
| **ScvO_2_ (%)** | Fluid removal started at initiation of CVVHDF (N=19) | 72 (71-74) | 72 (71-73) | 70 (69-72) | 70 (69-71) |
|  | Fluid removal started after initiation of CVVHDF (N=23) | 70 (68-75) | 71 (68-76) | 70 (67-70) | 70 (68-76) |
| **SBF (PU)** | Fluid removal started at initiation of CVVHDF (N=19) | 76 (41-125) | 43 (17-53)# | 35 (18-49)# | 40 (17-52)# |
|  | Fluid removal started after initiation of CVVHDF (N=23) | 98 (44-126) | 41 (19-103)# | 43 (19-86)# | 47 (19-71)# |

Data are expressed as medians with 25th and 75th percentile, *p<0.05 between patients in whom fluid removal was started at initiation vs later, #p<0.05 versus baseline (T0) value in the same group

Abbreviations: MAP-mean arterial pressure; CI-cardiac index; ScvO_2_-central venous oxygen saturation
